# Entscheidungshilfe zur Patientenzuweisung bei Verdacht auf COVID-19: Für die Anwendung im bayerischen Rettungsdienst

**DOI:** 10.1007/s10049-021-00855-3

**Published:** 2021-03-04

**Authors:** N. Thies, B. Urban, M. Kraus, T. Kohlmann, F. Niedermirtl, S. Prückner

**Affiliations:** 1 Institut für Notfallmedizin und Medizinmanagement (INM), Klinikum der Universität München, LMU München, München, Deutschland; 2 AG 4 des Rettungsdienstausschusses Bayern, Regierung von Unterfranken, Würzburg, Deutschland; 3 Rettungsdienstbereich Amberg, ZRF Amberg, Amberg, Deutschland

**Keywords:** Rettungsdienst, Notfallmedizin, Pandemie, Kategorisierung, SARS-CoV-2, Emergency medical service, Prehospital medical care, Pandemic, Categorization, SARS-CoV-2

## Abstract

Während einer Pandemielage sind Patienten mit Verdacht auf COVID-19 auch im Rahmen von Rettungsdienstalarmierungen zu versorgen. Um ein adäquates Vorgehen zu etablieren, ist die Entscheidungshilfe zur Patientenzuweisung bei Verdacht auf COVID-19 für die Rettungsdienstmitarbeiter sowie für die Notärzte im bayerischen Rettungsdienst erstellt worden. Die Entscheidungshilfe schließt die aktuellen Leitlinien und Empfehlungen zum Thema COVID-19 ein. Für die Darstellung der Entscheidungshilfe wurde ein Flussdiagramm in DIN-A4-Format gewählt, welches nach dem im Rettungsdienst etablierten ABCDE-Schema (A-Airway, B-Breathing, C-Circulation, D-Disability, E-Environment/Exposure)aufgebaut ist. Das Flussdiagramm ermöglicht eine Kategorisierung der Patienten in drei Stufen, welche anhand von (Vital‑)Parametern und Kriterien wie Risikofaktoren und spezifischen Rahmenbedingungen erfolgt. Ziel ist es, Notärzten und Rettungsdienstmitarbeitern eine Orientierungshilfe für die Beurteilung der Patienten sowie daraus entstehende Transportentscheidung mit gegebenenfalls geeigneter Zielklinik an die Hand zu geben.

## Abstract (en)

During a pandemic situation, patients with suspected coronavirus disease (COVID-19) are also treated by emergency medical services (EMS). In order to establish an adequate procedure, a decision aid for the allocation of patients in case of suspicion of COVID-19 has been prepared for the ambulance staff as well as for the emergency physician in the Bavarian EMS. The decision-making aid includes the current guidelines and recommendations on COVID-19. A flowchart in A4 format was chosen for the presentation of the decision aid, which is structured according to the ABCDE scheme (A-Airway, B-Breathing, C-Circulation, D-Disability, E-Environment/Exposure) established in EMS. The flowchart allows patients to be categorized in three stages, based on (vital) parameters and criteria such as risk factors and specific framework conditions. The aim is to provide emergency physicians and ambulance staff with guidance for the assessment of patients and the resulting transport decision with a suitable target clinic if necessary.

## Einleitung

Während der durch das Coronavirus SARS-CoV‑2 ausgelösten Pandemie besteht bei großen Teilen der Bevölkerung eine Verunsicherung, an COVID-19 erkrankt zu sein, wenn grippeähnliche Symptome wie Fieber, Husten oder Schnupfen auftreten. In diesem Zusammenhang kommt es in sehr unterschiedlichen Stadien der Erkrankung auch zur primären Alarmierung des Rettungsdienstes. Um eine angemessene Einweisungsstrategie in Kliniken für diese Patientengruppe sicherzustellen, müssen prozessuale Voraussetzungen erarbeitet werden.

Nach aktueller Studienlage zeigen COVID-19-Erkrankte in ca. 81 % einen milden, in ca. 14 % einen schweren und in ca. 5 % einen kritischen Verlauf [3, 7]. Eine der Herausforderungen für den Rettungsdienst ist es, die schwer und kritisch Erkrankten von denjenigen zu differenzieren, die ggf. auch im häuslichen Umfeld medizinisch versorgt werden können.

Dabei gilt es, die Ressourcen der Krankenhäuser nicht mit vermeidbaren Transporten und Krankenhausbehandlungen zu überlasten, damit diese für dringend behandlungsbedürftige Patienten in ausreichendem Maß zur Verfügung stehen. Parallel sollten Patienten identifiziert werden, die zwingend eine Zuweisung in ein Haus mit Intensiv- beziehungsweise Beatmungskapazität benötigen. Zu berücksichtigen ist, dass an die Entscheidung für eine ambulante Versorgung hohe Anforderungen zu stellen sind, da sich ein zunächst stabiler Zustand von COVID-19-Patienten auch rapide verschlechtern kann.

Zur Unterstützung des im Rettungsdienst tätigen Personals einschließlich der Notärztinnen und Notärzte wurde deshalb unter Berücksichtigung der aktuellen Literatur von einer interdisziplinär besetzten Arbeitsgruppe des Rettungsdienstausschusses (RDA) Bayern mit Unterstützung des Instituts für Notfallmedizin und Medizinmanagement (INM), LMU Klinikum, eine Entscheidungshilfe für die Zuweisung von Patienten mit klinischem Verdacht auf COVID-19 erarbeitet.

Ziel dieser Entscheidungshilfe ist es, eine Kategorisierung der präklinischen Patienten zu ermöglichen, um die zielgerichtete Zuweisung der Patienten in die geeignete Versorgungsstruktur zu kanalisieren.

## Entwicklung der Entscheidungshilfe

Zur Erstellung der Entscheidungshilfe wurden in einem ersten Schritt aktuelle Leitlinien, Empfehlungen sowie weitere Publikationen zum Thema COVID-19 gesichtet.

Eine Literaturrecherche ergab, dass viele Veröffentlichungen Orientierungshilfen für den ambulanten Sektor darstellen oder innerklinische Entscheidungsmechanismen beschreiben. Für den Rettungsdienst gibt es hingegen nur wenige Empfehlungen. Neue Scoring-Systeme in Bezug auf COVID-19 sind insbesondere für die Präklinik nicht validiert. Daher wurde die Suche neben der Datenbank PubMed® auch um aktuelle Empfehlungen von Institutionen, wie dem Robert Koch-Institut (RKI) und der World Health Organization (WHO), sowie um verfügbare Algorithmen von Rettungsdienstorganisationen außerhalb Deutschlands erweitert.

**Figure 1:**
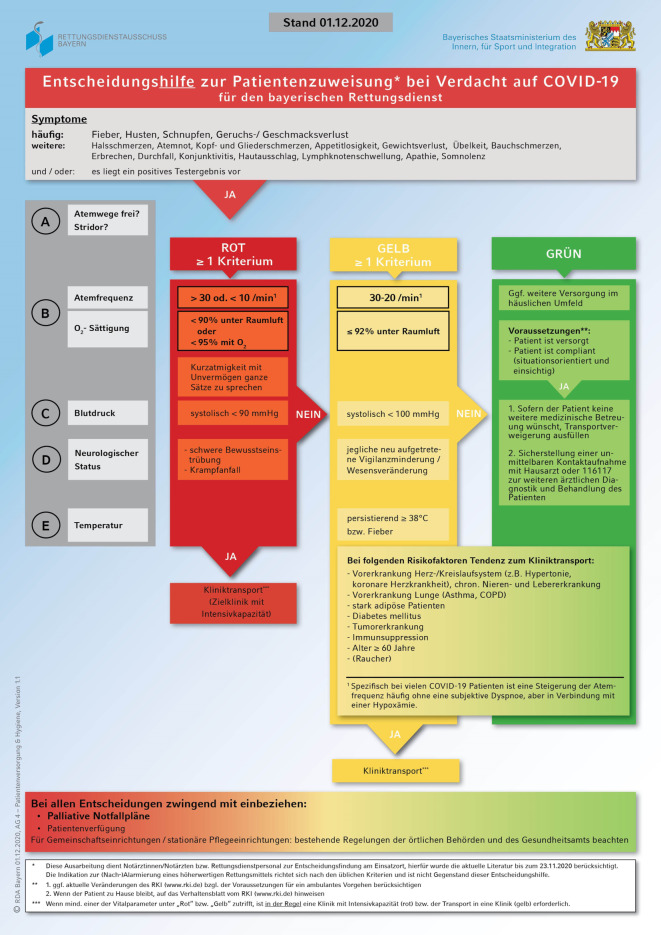

In einem weiteren Schritt wurde auf dieser Grundlage die Entscheidungshilfe für den bayerischen Rettungsdienst in Form eines Flussdiagramms erstellt, dabei wurde darauf Wert gelegt, dass der Inhalt übersichtlich bleibt und auf einer Seite darstellbar ist (siehe Abb. 1). Für einige Parameter werden beim Vergleich verschiedener Studien und Veröffentlichungen unterschiedliche Werte und Kriterien angegeben, sodass eine Festlegung auf konkrete Werte und Kriterien im Sinne eines Expertenkonsenses erfolgte.

Hierbei ist zu beachten, dass die Datenlage zu dieser Thematik rasch zunimmt und sich relevante Einschätzungen weiterentwickeln, sodass an manchen Stellen auf die jeweils aktuellen Empfehlungen der Institutionen verwiesen wird.

Das Flussdiagramm orientiert sich an dem für das Rettungsdienstpersonal in der täglichen Arbeit vertrauten ABCDE-Schema und soll eine Kategorisierung der Patienten ermöglichen.

Die Entscheidungshilfe stellt bewusst keine verbindliche Vorgabe für den bayerischen Rettungsdienst dar, sondern soll Notärzte und Rettungsdienstmitarbeiter bei der Entscheidungsfindung unterstützen und ihnen hierfür relevante Vitalparameter, Risikofaktoren und weitere zu berücksichtigende Punkte an die Hand geben.

## Details der Entscheidungshilfe

Eingangsvoraussetzung für die Abarbeitung des Flussdiagramms ist das Absetzen eines Notrufs, welcher nach Einschätzung des Disponenten der Integrierten Leitstelle die Alarmierung und Entsendung eines Notfallrettungsmittels notwendig macht. Treffen die Rettungsdienstmitarbeiter am Einsatzort auf einen Patienten, welcher typische Symptome einer COVID-19-Erkrankung aufweist, so wird auf Basis des Flussdiagramms die Entscheidungsfindung bzgl. eines Kliniktransports des Patienten oder einer Überleitung in die ambulante Betreuung unterstützt.

Typische Symptome für eine COVID-19-Erkrankung sind am Anfang des Flussdiagramms grob orientierend in „häufig“ (Fieber, Husten, Schnupfen und Geruchs‑/Geschmacksverlust) und „weitere Symptome“ (Halsschmerzen, Atemnot, Kopf- und Gliederschmerzen, Appetitlosigkeit, Gewichtsverlust, Übelkeit, Bauchschmerzen, Erbrechen, Durchfall, Konjunktivitis, Hautausschlag, Lymphknotenschwellung, Apathie, Somnolenz) unterteilt worden [8].

Um ein strukturiertes Vorgehen zu ermöglichen sowie ein im Rettungsdienst etabliertes Schema zu nutzen, ist die Entscheidungshilfe nach dem ABCDE-Schema (A – Airway, B – Breathing, C – Circulation, D – Disability, E – Environment/Exposure) aufgebaut. In dem ABCDE-Schema werden die zu prüfenden Parameter für die Zuweisungsentscheidung konkret genannt. Dabei ist zu berücksichtigen, dass nicht alle Parameter des ABCDE-Schemas aufgeführt werden, sondern nur die relevanten Parameter für die Entscheidung bei einem COVID-19-Verdachtsfall. Anhand der ermittelten Werte kann eine Einteilung in die Kategorien rot, gelb und grün vorgenommen werden. Die Struktur des Flussdiagramms sieht vor, zunächst die Kriterien der roten Kategorie, dann der gelben und zum Schluss der grünen durchzugehen. Sobald ein Kriterium erfüllt ist, führt dies zum Verbleib des Patienten in dieser Kategorie. Jeder Kategorie ist eine Empfehlung zum Kliniktransport oder ambulanten Vorgehen zugeordnet.

Bei „rot“ kategorisierten Patienten wird als Zielklinik ein Haus mit freier Intensivkapazität empfohlen, bei „gelb“ eingestuften Patienten sollte auf jeden Fall ein Transport in eine Klinik durchgeführt werden. Erfüllt der Patient die Kriterien der grünen Kategorie mit den Voraussetzungen für eine ambulante Behandlung, kann ein Transportverzicht erwogen werden.

Die Kriterien für die rote Kategorie basieren auf Empfehlungen für eine intensivmedizinische Behandlung, die maßgeblich durch erhöhte Atemfrequenz > 30/min, Hypoxämie sowie Dyspnoe beschrieben werden [3, 4, 9, 10]. Dies unterstützt auch das Ergebnis von Fistera et al., die im Rahmen einer Validierung eines Modells zur sicheren und effizienten Triage an der Uniklinik Essen feststellten, dass Patienten mit einem auffälligen CT und normalen Vitalparametern nach Hause entlassen werden konnten, während Patienten mit einer erhöhten Atemfrequenz, einer reduzierten Sättigung oder einer Ruhedyspnoe stationär überwacht werden mussten [2]. Klinisch wird häufig eine kompensatorische Steigerung der Atemfrequenz beschrieben, ohne dass der Patient eine Dyspnoe empfindet. Dieses Phänomen ist auch unter dem Begriff „silent hypoxia“ bekannt und unterstreicht die Bedeutung der Atemfrequenz [6]. Ebenso spielen Kriterien wie eine Hypotonie oder ein pathologischer neurologischer Status bei der Zuordnung zur roten Kategorie eine Rolle.

Die gelbe Kategorie erfasst Kriterien für Patienten mit einem deutlich erhöhten Risiko für einen potenziell kritischen Verlauf einer COVID-19-Erkrankung, die oft eine stationäre Behandlung erforderlich machen [2, 5, 11–15]. Die für diese Kategorie in der Entscheidungshilfe vermerkten Kriterien sind jedoch nicht dafür entwickelt, von vorneherein eine stationäre Aufnahme oder einen Rettungsdiensteinsatz zu initiieren. Vielmehr haben diverse begleitende Gründe den Anrufer und den ILS-Disponenten bereits veranlasst, ein Notfallrettungsmittel anzufordern bzw. zu entsenden. In einer solchen Situation sind an einen Transportverzicht auch aus forensischen Gründen hohe Anforderungen zu stellen. Nach einer bereits erfolgten Alarmierung eines Notfallrettungsmittels profitiert diese Patientengruppe von einer Vorstellung in einer Klinik/Notaufnahme, um dort unmittelbar die empfohlene erweiterte Diagnostik durchführen zu lassen, die präklinisch nicht zur Verfügung steht (Labor, Sonographie, radiologische Verfahren etc.). Nach entsprechender Komplettierung der Diagnostik muss dann dort die endgültige Reevaluation der Kategorisierung erfolgen.

Die Voraussetzungen für eine ambulante Behandlung und weitere häusliche Versorgung (grüne Kategorie) gehen aus den RKI-Empfehlungen für ein ambulantes Vorgehen hervor [16]. Die Kernpunkte sind in das Schema aufgenommen. Sollten die Voraussetzungen für eine weitere Versorgung im häuslichen Umfeld erfüllt sein und der Rettungsdienst sich entscheiden, den Patienten zu Hause zu lassen, ist sicherzustellen, dass die weitere Diagnostik und Behandlung durch den Hausarzt oder den Ärztlichen Bereitschaftsdienst der Kassenärztlichen Vereinigung Bayerns (erreichbar unter der Telefonnummer 116117) zeitnah gewährleistet sind. Vom weiterbehandelnden Arzt müssen die Voraussetzungen zur weiteren ambulanten Versorgung erneut evaluiert werden. Dieser ist zuständig für das weitere Vorgehen und kann dabei auch im weiteren Verlauf bei Erfordernis eine Klinikeinweisung organisieren. Entscheidend ist, dass eine weitere ambulante ärztliche Betreuung stattfindet, da aktuelle Erkenntnisse zeigen, dass es häufig erst am 7–10 Tag nach Symptombeginn zu einer pulmonalen Verschlechterung der Symptomatik mit einer Hypoxämie kommt [1, 6, 17]. Zudem soll der Patient auf die Empfehlungen des RKI „*zum ambulanten Management von COVID-19-Verdachtsfällen und leicht erkrankten bestätigten COVID-19 Patienten*“ hingewiesen werden [16].

Die angegebenen Parameter zur Entscheidungsfindung können nur eine Orientierungshilfe sein. Insgesamt ist bei der Beurteilung der angegebenen Grenzwerte für Vitalparameter zu berücksichtigen, ob diese im individuellen Fall physiologisch oder durch bekannte Vorerkrankungen zu erklären sind und gegebenenfalls nicht zwingend im Zusammenhang mit einer COVID-19-Erkrankung stehen.

Wichtig ist auch die Einbeziehung von Patientenwillen, palliativen Notfallplänen und Patientenverfügungen, soweit vorhanden. Weiterhin sind insbesondere bei Gemeinschaftsunterkünften und stationären Pflegeeinrichtungen bei einer Pandemie seitens der zuständigen örtlichen Behörden, vor allem des Gesundheitsamts, oft spezielle Regelungen und Versorgungskonzepte etabliert, die beachtet und in die Überlegungen bezüglich einer Entscheidung zur ambulanten Versorgung einbezogen werden müssen.

Außerdem kann die Entscheidung natürlich vor Ort situationsadaptiert angepasst werden. Dies kann die Kategorie, aber auch die Entscheidung des Transportziels und die Entscheidung für ein ambulantes Vorgehen betreffen.

## Diskussion

Das vorliegende Flussdiagramm soll Rettungsdienstpersonal und Notärzte bei Patienten mit Verdacht auf COVID-19 bei der Auswahl der geeigneten Zielklinik unterstützen, aber auch als Entscheidungshilfe für eine weitere Versorgung im häuslichen Umfeld dienen.

Ziel muss in der Pandemie einerseits die Vermeidung einer Übertriagierung sein, um wertvolle Ressourcen der Kliniken während einer Pandemie nicht unnötig in Anspruch zu nehmen, damit diese dringend stationär behandlungsbedürftigen Patienten zur Verfügung stehen. Andererseits muss aber auch eine Untertriagierung vermieden werden, weshalb gerade bei den Vitalparametern die Grenzwerte, die eine Vorstellung in einer Klinik triggern, tendenziell konservativ gewählt wurden. Weiterhin basiert die Entscheidungshilfe auf Kriterien, die im Rettungsdienst mit einfachen Mitteln zu erfassen und eindeutig sind. Zusätzlich geeignete Untersuchungen wie Sonographie und POCT-Diagnostik zu D‑Dimeren wurden nicht berücksichtigt, da diese in Bayern präklinisch kaum verfügbar sind. Dies gilt auch für Kriterien, die in klinischen COVID-19-Scores einbezogen werden, aber im präklinischen Setting nicht erfasst werden können, wie beispielsweise weitere Laborwerte. Parameter, die nur in wenigen Publikationen genannt sind, wie z. B. der diastolische Blutdruckgrenzwert, und Kriterien, die keine eindeutigen Grenzwerte beinhalten und dadurch einen zu großen Diskussionsspielraum haben, wurden ebenfalls nicht aufgenommen.

Ein höheres Lebensalter wird in nahezu allen Publikationen/Leitlinien bei den Risikofaktoren für einen schwereren Verlauf aufgeführt. Allerdings gibt es keinen Konsens bezüglich einer exakten Altersangabe oder einer Altersspanne. Die Altersangaben, die veröffentlicht sind, beziehen eine Altersspanne von > 50 Jahren bis > 65 Jahren ein [8, 13, 15, 17]. Auf dieser Grundlage wurde als Expertenkonsens ein Risikoalter ≥ 60 Jahre festgelegt.

Berücksichtigt wurden zudem Empfehlungen zur Abschätzung einer Klinikeinweisung unabhängig von COVID-19-Erkrankungen. Als Hilfe zur Entscheidung bezüglich eines Kliniktransports eines Patienten mit Pneumonie wurde der CRB-65-Index herangezogen. Dieser Score wird von der Deutschen Gesellschaft für Allgemeinmedizin und Familienmedizin (DEGAM) auch als Entscheidungshilfe bei COVID-19-Patienten empfohlen, da es darüber hinaus keine validierten Scores mit Grenzwerten gibt [12].

Welcher Patient bei einem Verdacht auf eine Infektion ein erhöhtes Letalitätsrisiko hat und damit eine hohe Wahrscheinlichkeit für einen Intensivaufenthalt durch ein zunehmendes Organversagen aufweist, kann auch mit dem qSOFA-Score detektiert werden. Der Leitfaden zur „ambulanten patienten-zentrierten Vorausplanung für den Notfall“, welcher von mehreren medizinischen Fachgesellschaften (unter anderem der Deutschen Gesellschaft für Anästhesiologie und Intensivmedizin [DGAI]) veröffentlicht wurde, empfiehlt, bei der medizinischen Indikationsstellung einer Krankenhauseinweisung den qSOFA-Score zu berücksichtigen [14]. Bei COVID-19-Erkrankungen soll der qSOFA-Score noch um das Kriterium der Sauerstoffsättigung ergänzt werden. Dies geht auf eine Empfehlung der Deutschen Gesellschaft für Pneumologie zurück [4]. In einer Publikation von Breuer et al. wird eine niedrige Sauerstoffsättigung bei Klinikaufnahme mit einem kritischen Verlauf assoziiert [1]. Die Mitberücksichtigung des qSOFA-Scores wird auch von der WHO empfohlen, welche ein Risiko für eine erhöhte Letalität bei Patienten mit höherem Lebensalter, einem höheren SOFA-Score sowie erhöhten D‑Dimeren bei der Aufnahme beschreibt [9]. Mit dem vorgestellten Flussdiagramm kann sowohl das Vorliegen der Kriterien des CRB-65-Index als auch des erweiterten qSOFA-Scores bei den Patienten abgebildet und so die Entscheidung für einen Transport in die Klinik unterstützt werden.

Auch wegen rückläufiger COVID-19-Fallzahlen in den Sommermonaten und damit verbundenem geringerem Einsatzaufkommen des Rettungsdiensts mit COVID-19-Verdachtspatienten konnte noch keine Validierung der „Entscheidungshilfe zur Patientenzuweisung bei Verdacht auf COVID-19 für den bayerischen Rettungsdienst“ durchgeführt werden. Durch die aktuell steigenden Fallzahlen und dadurch höheren Kontaktzahlen mit dem Erkrankungsbild im Rettungsdienst wird aktuell eine Validierung angestrebt.

Anfang November hat eine Überarbeitung der Entscheidungshilfe zur Patientenzuweisung bei Verdacht auf COVID-19 für den bayerischen Rettungsdienst unter Berücksichtigung der aktuellen Literatur stattgefunden.

## Fazit für die Praxis

Der Algorithmus dient als orientierende Hilfestellung bei der Beurteilung von Patienten mit Verdacht auf COVID-19 im Rettungsdiensteinsatz. In dieser Entscheidungshilfe für Rettungsdienstpersonal und Notärzte sind nur Kriterien berücksichtigt, die auch präklinisch erhoben werden können. Bei einer Klinikeinweisung muss die Kategorie nochmals mit den dort erweiterten Möglichkeiten der Diagnostik reevaluiert werden.
